# Hospital Meetings, &c.

**Published:** 1898-02-12

**Authors:** 


### HOSPITAL MEETINGS, &C.

### THE LONDON HOSPITAL.

The twenty-third anniversary dinner of the General East End Tradesmen's Association in aid of the London Hospital, Whitechapel, of which the Duke of Cambridge is president, was held on Thursday night at the London Tavern, Fenchurch Street. Mr. SPENCER CHARRINGTON, M.P., presided, and was supported by the Hon. Sydney Holland, Mr. H. L. W. Lawson, Sir William Porter, Mr. E. Wynne Baxter, Mr. G. Q. Roberts, and Mr. John Loftus.

The CHAIRMAN, in proposing "Success to the London Hospital," alluded to the growing demands made on the resources of this and other hospitals in the metropolis, and said he thought the time was bound to come when the State would be called upon to make a grant to such important institutions.

Mr. LAWSON supported the toast. He said the London Hospital had only one operating theatre, although last year they had no fewer than 2,196 of what were called major operations, and 60 cases each week in the wards which required anæsthetics. As a consequence, poor people were day after day prepared for operations which it was often found impossible to carry out owing to lack of accommodation. He concluded by expressing his disagreement with the view of the chairman that our hospitals should be maintained out of the rates and taxes.

The Hon. SYDNEY HOLLAND, chairman of the hospital, said the London Hospital was at the present time in a very serious condition. They required £70,000 a year to keep it going, of which only £20,000 was obtained from subscriptions and donations. In the last two years they had had to sell £30,000 of their investments to meet their deficit, but, of course, that sort of thing could not go on for ever. He had no hesitation in saying that the Prince of Wales's Fund had saved the London hospitals. They were to receive from that Fund £5,000 a year, which would render it possible for them to sell £100,000 of their investments, and with the proceeds make certain necessary improvements to the hospitals. He did not want the hospitals to be supported out of rates, but he certainly thought that the Government should contribute towards the expense incurred by them in training medical men. In conclusion, he said that unless the work of the London Hospital was to be curtailed they must have at least an additional £10,000 a year.

Other toasts followed, and during the evening Mr. J. B. Caldow, treasurer of the association, was presented with a timepiece in recognition of his valuable services.

### CITY OF LONDON TRUSS SOCIETY.

The annual meeting of the supporters of this charity was held on Wednesday, 2nd inst., at the offices, 35, Finsbury Square, Mr. C. BARHAM, C.C., presiding. The report stated that the gross income was £5,551, as against £4,549 in 1896. The expenditure was £5,907. The number of patients relieved was 9,601, to whom 9,663 instruments were supplied. The Chairman, in moving the adoption of the report, said that, during the many years he had been connected with the society he had never known a more satisfactory report than that placed in the subscribers' hands that day. Votes of thanks and the election of the committee and other officers concluded the proceedings.

